# Breaking the Silence: Exploring Barriers to Raising Concerns in Psychiatry

**DOI:** 10.1192/bjo.2025.10318

**Published:** 2025-06-20

**Authors:** Megan Tymanskyj, Sabrina Hasnaoui, Melissa O’Conner-Smith, Cammeron Meades, Joseph Farmer

**Affiliations:** Coventry and Warwickshire Partnership Trust, Coventry, United Kingdom

## Abstract

**Aims:** Raising concerns is an important duty for those working in medicine, which can have a broad impact on factors including safety, training, and wellbeing. This project aims to explore resident doctors’ experiences of raising concerns in psychiatry, including establishing awareness of available processes, and identifying barriers to utilising these. This work has been conducted as part of a wider Quality Improvement Project, aiming to improve resident doctor awareness and engagement with the process of raising concerns by overcoming identified barriers.

**Methods:** Resident doctors of various grades working in psychiatry within a six month period were invited to attend focus groups to gather information about their perspectives of raising concerns. Thematic analysis of focus group discussion was conducted. Quantitative data was obtained from an online survey which was sent to all resident doctors working in the trust for anonymous completion.

**Results:** 19 resident doctors attended focus groups. Thematic analysis of this content demonstrated five key themes with additional subthemes:

Repercussions (impact on career + feedback, wellbeing, reputation).

Futility.

Uncertainty (culture, acceptability, process).

Division (hierarchy, staff groups).

Variability (receptiveness, response, supervisor relationship).

25 resident doctors responded to the survey: 52% felt unfamiliar with the process for raising concerns; 5 respondents had raised a concern within the trust; 9 had experienced concerns that they had wanted to raise but could not.

Most concerns related to training (56%), supervision from seniors (31%), patient safety (25%), bullying/harassment (19%), and resident doctor wellbeing (13%). 16% of respondents felt that a barrier to raising a concern was related to race, sexuality, gender, or any other protected characteristic. 57% felt they were not taken seriously when they had raised a concern. 71% felt they had not received adequate feedback after raising a concern.

**Conclusion:** Resident doctors are experiencing a range of concerns, but many find that barriers prevent them from raising these. These barriers generally relate to uncertainty regarding the process, futility of raising concerns based on previous experiences, and fear of repercussions. This data suggests that there are issues resident doctors are experiencing that are going unreported, relating to their own training experiences as well as patient safety concerns. Focus group data has allowed us to have a better understanding of the barriers resident doctors face when raising concerns. Subsequently, we are working alongside resident doctors and key stakeholders using Quality Improvement methodology to trial the implementation of several change ideas to streamline the process of raising concerns.

